# The anticancer effect of chaetocin is enhanced by inhibition of autophagy

**DOI:** 10.1038/cddis.2016.15

**Published:** 2016-02-18

**Authors:** H-J Jung, I Seo, F Casciello, S Jacquelin, S W Lane, S-Il Suh, M-H Suh, J S Lee, W-K Baek

**Affiliations:** 1 Department of Microbiology, School of Medicine, Keimyung University, Daegu, Republic of Korea; 2 Control of Gene Expression Laboratory, QIMR Berghofer Medical Research Institute, Herston, Queensland, Australia; 3 Division of Immunology, QIMR Berghofer Medical Research Institute, Herston, Queensland, Australia; 4 Institute for Cancer Research, Keimyung University, Daegu, Republic of Korea

## Abstract

Chaetocin is a fungal metabolite that possesses a potent antiproliferative activity in solid tumors by inducing cell death. Although recent studies have extended the role of chaetocin in tumors, the underlying molecular mechanisms such as the downstream cascade that induces cell death has not clearly been elucidated. In this study, we show that chaetocin is able to induce both apoptosis and autophagy in several hepatoma cell lines including HepG2, Hep3B and Huh7 cell lines. Moreover, we found that the inhibition of caspase-3/7 activity by z-VAD-fmk treatment was able to block chaetocin-mediated cell death, whereas blocking autophagy by Bafilomycin A1 or the knockdown of autophagy protein 5 enhanced cell death mediated by chaetocin. These findings suggest that chaetocin has a potent anticancer effect against hepatoma. Inhibition of autophagy may potentiate anticancer effects of chaetocin thus providing evidence that combined treatment with chaetocin and autophagy inhibitors will be an effective strategy for treating cancer.

Chaetocin, is a natural substance produced as a fungal metabolite,^1, 2^ with its chemical structure defined to be belonging to the diketoepiperazines.^3^ It was discovered as a potent and selective anti-myeloma agent as it induced cellular oxidative stress.^4^ Chaetocin has since been tested in a broad range of cancer cell lines and potently inhibit proliferation in solid tumors by inducing proinflammatory response and cell death pathways.^5^ Chaetocin appears to have a multiple role in cancer cells as it was able to induce not only cellular oxidative stress, but also apoptosis. Moreover, chaetocin may not only target tumor cells directly, but also indirectly inhibit tumor growth by reducing angiogenesis at the tumor microvasculature level. More recently, chaetocin has received further attention as it was able to inhibit HIF-1*α* signaling by inhibiting the transactivation potential of HIF-1*α* by attenuating its binding to p300, and thereby inhibiting the growth of HepG2 xenograft.^6^ The inhibitory role of chaetocin on tumor growth was further demonstrated in another study, which showed that the effect of chaetocin on tumor growth required HIF-1*α*^7^ and thus a potential agent to be used for solid tumors.

Chaetocin has also emerged as an epigenetic agent affecting the methylation status of histone H3K9 methylation by inhibiting histone lysine methyltransferase SU(VAR)3-9 in *Drosophila,*^8^ while it was reported to be a nonspecific inhibitor of histone lysine methyltransferases^9^ particularly toward SUV39h1 to ultimately affect gene expression. Elevated SUV39h1 expression and high level of H3K9me3 resulting from the change in the methyltransferase expression affected the development and progression of hepatocellular carcinoma (HCC).^10^ ROS-mediated apoptosis appears to be the mechanism by which chaetocin acts as an anticancer agent in glioblastoma model.^11^ As oxidative stress is an important regulator of apoptosis and metabolic reprogramming, both pathways were affected by chaetocin treatment. Moreover, chaetocin treatment ultimately caused a reduction in tumor burden in glioma xenografts suggesting its potential use as a novel therapeutic agent.

Autophagy is involved in removing and recycling of micro-organelles and abnormal proteins. Autophagy can be induced by nutrient-depriving states and serves as a cell survival response.^12^ It is becoming more evident that autophagy can lower the efficacy of anticancer agents by enhancing cellular survival in stressed conditions elicited by cancer therapeutic agents.^13, 14^ In the article, we report that chaetocin is able to induce both apoptosis and autophagy in human hepatoma cell lines, and suppression of autophagy enhances caspase-dependent apoptotic cell death. Based on these results, chaetocin may be an effective agent to be used in combination with pharmacological inhibitors of autophagy.

## Results

### Chaetocin induces cell death in human hepatoma cell lines

**Figure 1:**
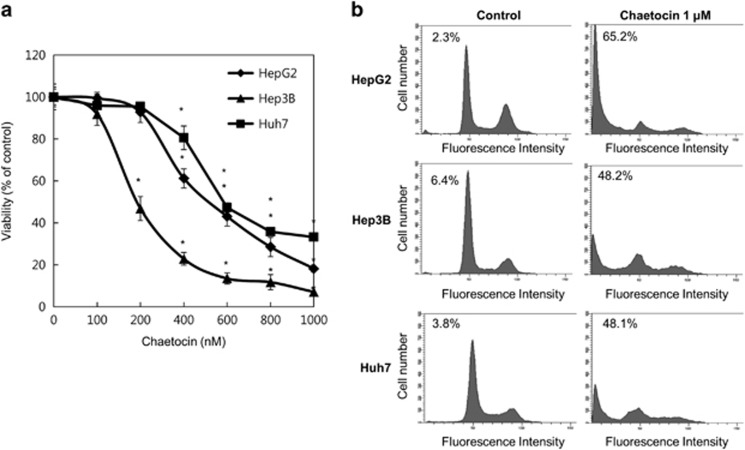
Chaetocin induces cell death in human hepatoma cell lines. (**a**) Viability of three human hepatoma cell lines (HepG2, Hep3B and Huh7) following chaetocin treatment is shown. Cells were treated with various doses of chaetocin as indicated for 24 h and cell viability was determine by cell counting with Trypan blue staining. Data are expressed as % of control±S.E.M. for pooled data from three experiments, each performed in triplicate (*n*=3, *P<0.05). (**b**) The hepatoma cell lines were treated with either vehicle control or chaetocin (1 *μ*M) for 24 h and subG1 population was determined by flow cytometric analysis following PI staining. Indicated is the percentage of subG1 population in each cell line (*n*=3)

Various hepatoma cell lines were used to test the cytotoxic effect of chaetocin. Upon treatment of cells with increasing concentrations of chaetocin for 24 h, the viability of all three cell lines was significantly reduced by chaetocin (Figure 1a). The ED50 of chaetocin for HepG2, Hep3B and Huh7 were 545.254, 253.388 and 677.046 nM, respectively, with Hep3B being the most sensitive cell line to chaetocin.

Next, in order to determine the mode of cell death by chaetocin, cells were treated with 1 *μ*M chaetocin for 24 h and harvested for cell cycle distribution analysis using flow cytometry. It was clear that chaetocin treatment resulted in a marked increase in the subG1 population 65.2% compared with 2.3% in the control HepG2 cells, which was indicative of apoptosis (Figure 1b). The proapoptotic effect of chaetocin was also observed in the Huh7 and Hep3B cell lines with >12- and 7-fold increases in the subG1 population compared with control cells, respectively (Figure 1b). The ability of chaetocin to elicit apoptosis was verified by a poly ADP ribose polymerase (PARP) cleavage assay (Supplementary Figure S1). HepG2 cells were pretreated with a caspase-3 inhibitor (z-VAD-fmk) for 30 min followed by chaetocin for further 24 h. Chaetocin caused a significant increase in cleavage of PARP, whereas the pretreatment of z-VAD-fmk inhibited this cleavage. Upon examining for cell viability, z-VAD-fmk treatment was able to protect cells from chaetocin-mediated cell death.

### Chaetocin causes an increase in apoptosis markers in a time- and dose-dependent manner

**Figure 2:**
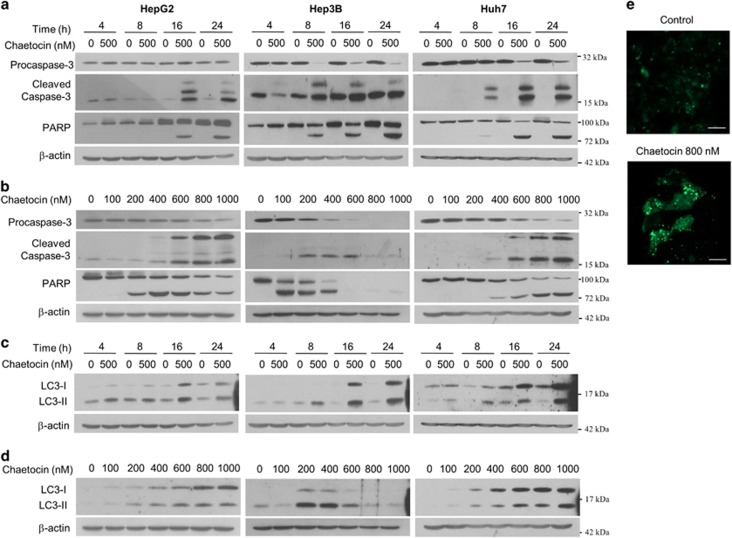
Chaetocin causes an increase in apoptosis markers in a time- and dose-dependent manner. (**a** and **b**) Immunoblotting of procaspase-3, cleaved caspase-3 and PARP from three hepatoma cell lines following different treatment times (**a**) and various doses (**b**) of chaetocin as indicated. Immunoblotting for *β*-actin was used as loading control. (**c** and **d**) Immunoblotting of autophagy marker; LC3 from three hepatoma cell lines following different treatment times (**c**) and various doses (**d**) for chaetocin. (**e**) Immunofluorescence analysis of GFP-LC3 in HepG2 cells with or without chaetocin treatment (800 nM) for 24 h. Images are shown at × 400 magnification. Scale bar, 20 *μ*m

We next examined the effect of chaetocin treatment on the induction of apoptotic response. HepG2, Hep3B and Huh7 cells were treated with 500 nM of chaetocin for 4 to 24 h (Figure 2a). The cleavage of both caspase-3 and PARP was visible as early as 4 h of chaetocin treatment in Hep3B and Huh7 cells, and by 16 h in HepG2 cells. Cells treated with increasing concentration of chaetocin (0–1000 nM) showed a dose-dependent increases in the cleavage of caspase-3 and PARP (Figure 2b). Together, these data indicate that chaetocin can induce an apoptotic response in hepatoma cell lines in a time- and dose-dependent manner.

Next, we examined whether chaetocin induces autophagy. We treated three human hepatoma cell lines with chaetocin and examined for autophagy marker, microtubule-associated protein light chain 3-II (LC3-II). Indeed, chaetocin was also able to induce LC3-II in all cell lines tested in a time- and dose-dependent manner (Figures 2c and d). In order to confirm the induction of autophagy, GFP-LC3-transfected HepG2 cells were incubated in the absence and presence of chaetocin for 24 h (800 nM) and immunofluorescence imaging was performed for LC3 expression. Chaetocin treatment was able to markedly induce the expression of GFP-LC3 puncta, which was indicative of an autophagosome formation (Figure 2e). Upon examining cell lines other than hepatoma origin, it was clear that chaetocin was able to induce LC3-II induction. Both T98G and U251MG glioma cell lines, as well as breast cancer cell lines such as MCF-7 and MDA-MB-231 showed a marked increase in LC3-II level following a chaetocin treatment (Supplementary Figure S2). Taken together, chaetocin appears to be inducing both apoptosis and autophagy in a variety of cancer cell lines including HCC cells.

### Chaetocin induces autophagic flux

**Figure 3:**
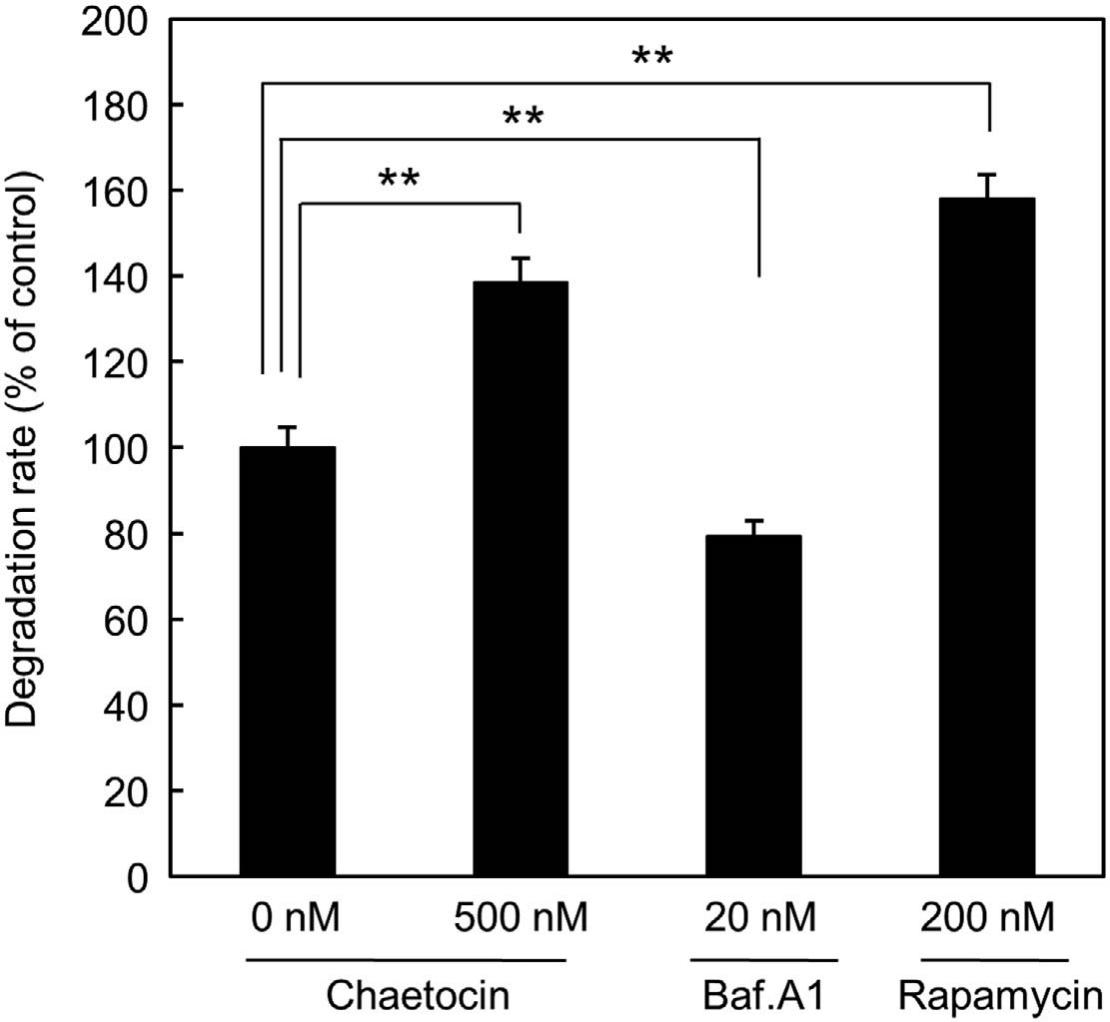
Chaetocin induces autophagic flux. The degradation rate of long-lived proteins following treatment with chaetocin, Baf.A1 and rapamycin for 4 h. Shown are average of triplicate samples analyzed and asterisks indicate *P*-value <0.005

Next, the autophagic degradation was examined to determine whether chaetocin-mediated induction of LC3-II expression and the accumulation of autophagosome were caused by the activation of autophagy or inhibition of autophagic degradation. Early autophagic markers such as LC3-II and punctated GFP-LC3 were elevated by both activation and suppression of autophagy. Disturbance of the late-phase autophagic processes like the autophagosome–lysosome fusion, the enzymatic degradations in the lysosome can also results in the accumulation of LC3-II protein and punctated GFP-LC3 in cells. To ascertain the cause of LC3-II protein accumulation and punctated GFP-LC3, autophagic degradation assay was performed in HepG2 cells using a ^14^C isotope. Chaetocin significantly increased degradation in a similar manner to an autophagy inducer, rapamycin (used as positive control for autophagy induction), whereas the autophagy inhibitor Bafilomycin A1 (Baf.A1) significantly reduced degradation compared with control (Figure 3).

### Suppression of autophagy enhances chaetocin-induced cell death

**Figure 4:**
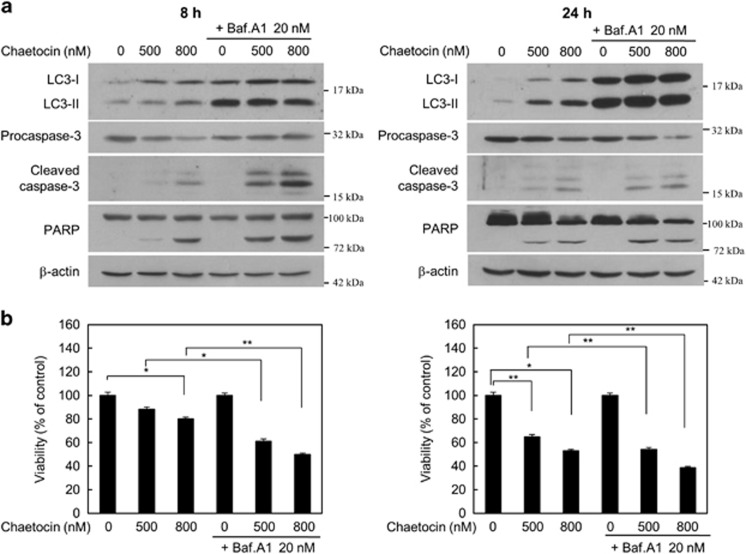
Suppression of autophagy enhances chaetocin-induced cell death. (**a**) Immunoblotting of LC3, procaspase-3, cleaved caspase-3 and PARP from HepG2 cells treated with chaetocin in the absence or presence of Baf.A1 for 8 h or 24 h. Immunoblotting forβ-actin was used as loading control. (**b**) Cell viability assay on HepG2 cells following the treatments as described in (**a**). Data are expressed % of control±S.E.M. for from triplicate samples (*n*=3, *P<0.05, **P<0.005, by ANOVA)

In order to determine whether suppression of autophagy results in enhancing chaetocin-induced cell death, we next examined the effect of Baf.A1 on chaetocin-induced apoptosis and autophagy. Cells treated with chaetocin for 8 or 24 h not only resulted in a marked increase in LC3-II levels but also induced caspase-3 and PARP cleavage. Upon co-treatment with Baf.A1, the protein level of LC3-II was further increased by inhibiting the autophagic flux. Moreover, further induction of apoptotic markers such as the cleavage of caspase-3 and PARP was evident with Baf.A1 treatment (Figure 4a). Consistent with the change in both apoptotic and autophagic markers, there was a significant reduction in cell viability when HepG2 cells were treated with chaetocin in combination with Baf.A1 (Figure 4b).

### Chaetocin-induced apoptosis is enhanced by inhibiting autophagy

**Figure 5:**
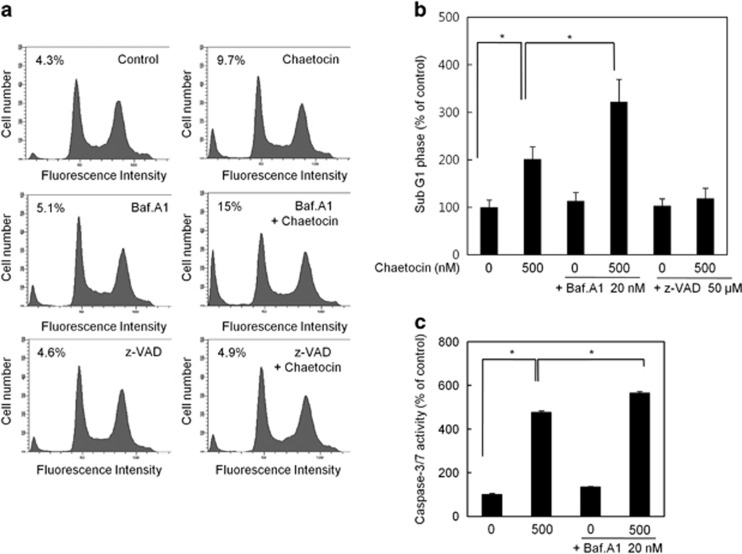
Chaetocin-induced apoptosis is enhanced by inhibiting autophagy. (**a**) Flow cytometric analysis of HepG2 cells treated with either chaetocin alone or in combination with Baf.A1 or z-VAD-fmk for 8 h showing the percentages of subG1 population as indicated. (**b**) Cell population in sub G1 phase from flow cytometric analysis performed in (**a**) is shown. Data are expressed as mean percentage of control±S.E.M. for pooled data from three independent experiments (Figure 5a and Supplementary Figure S3). (**c**) Caspase-3/7 activity assay of HepG2 cells treated as described in **a**. Shown are average of triplicate samples analyzed (*n*=3) from three independent experiments and asterisks indicate *P*-value <0.05

To gain further insight into how chaetocin elicits apoptotic cell death, fluorescence-activated cell sorting (FACS) analysis was performed using a propidium iodide (PI) staining to quantitate the apoptotic population (subG1) when treated with chaetocin alone, and co-treated with either Baf.A1 or the caspase inhibitor; z-VAD-fmk for 8 h (Figure 5a and Supplementary Figure S3). There was a >2-fold increase in the apoptotic population when cells were treated with chaetocin (9.7%) compared with control (4.3%). This increase in apoptotic population by chaetocin was further elevated by the co-treatment with Baf.A1, leading to 15% of the cell population being apoptotic. However, chaetocin-mediated induction of apoptosis was almost completely blocked by z-VAD-fmk treatment, suggesting that the activation of apoptosis by chaetocin is caspase dependent (Figure 5b). The effect of Baf.A1 on enhancing apoptosis was further confirmed by examining the caspase-3/7 activity (Figure 5c). Consistent with FACS analysis, it was clear that the induction of caspase-3/7 activity by chaetocin was significantly enhanced by Baf.A1 treatment.

### Loss of ATG5 enhances chaetocin-induced cell death

**Figure 6:**
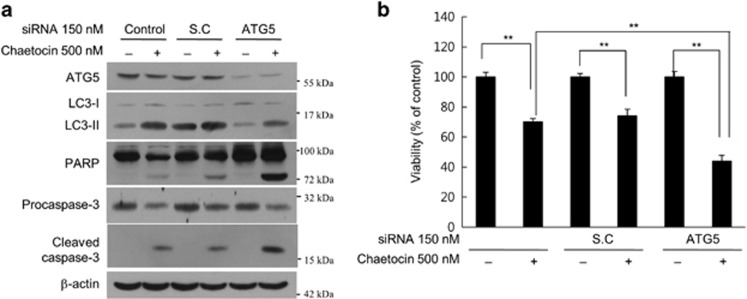
Loss of ATG5 enhances chaetocin-induced cell death. (**a**) Immunoblotting of autophagic markers (ATG5 and LC3-II) and apoptotic markers (PARP, cleaved caspase-3 and procaspase-3) from HepG2 cells transfected with the indicated siRNAs followed by chaetocin treatment for 16 h. (**b**) Cell viability assay on HepG2 cells following the treatments as described in (**a**). Shown are average of triplicate samples analyzed (*n*=3) and asterisks indicate *P*-value <0.005

An important question was whether the apoptosis enhancement by Baf.A1 in chaetocin-treated cells was due to the blocking of autophagy. To address this question, caspase-3 and PARP cleavage was examined in cells transfected with either scrambled small interfering RNA (siRNA; S.C) or autophagy protein 5 (ATG5) siRNA in the presence and absence of chaetocin (Figure 6). Chaetocin-mediated induction of LC3-II was abrogated upon knockdown of ATG5, whereas the cleavage of caspase-3 and PARP was moderately enhanced (Figure 6a). This increase in the activation of apoptosis was reflected upon cell viability as chaetocin treatment mildly reduced cell viability to 85.3% in cells transfected with scrambled siRNA (S.C), whereas cell viability was significantly reduced to 70% with ATG5 knockdown (Figure 6b). Chaetocin treatment in the absence of ATG5 led to a complete inhibition of LC3-II induction and markedly elevated levels of cleaved caspase-3 was observed which was associated with significant reduction in cell viability (Supplementary Figure S4). Together, these results strongly suggest that chaetocin-induced cell death can be enhanced by blocking autophagic pathway.

### Suppression of autophagy enhances inhibitory effects of chaetocin on colony formation

**Figure 7:**
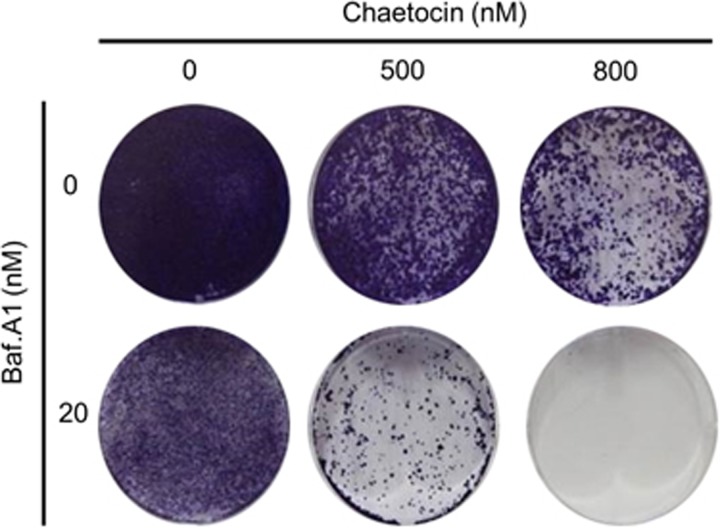
Suppression of autophagy enhances chaetocin-induced cell death. Crystal violet staining of colonies formed from HepG2 cells treated with chaetocin in the absence and presence of Baf.A1 as shown

We next investigated the effect of chaetocin on colony formation of HepG2 cells. Cells were treated with 500 and 800 nM for 24 h and grown for 2 weeks with growth media changed every 2–3 days (Figure 7). Crystal violet staining revealed that chaetocin had a profound impact on colony formation. The number of colonies formed from cells treated with chaetocin was significantly lower compared with that of vehicle-treated cells. Moreover, the inhibitory effect of chaetocin on colony formation was dose dependent as the number of colonies formed from cells treated with 800 nM chaetocin was markedly lower than that of 500 nM treatment. Further, the co-treatment of cells with Baf.A1 potentiated the effect of chaetocin's ability to reduce the formation of colonies such that no colonies developed from cells treated with both chaetocin (800 nM) and Baf.A1. These data suggested that autophagy induced by chaetocin does not appear to depend on processes that mediate cell death, but those related to cell survival. Therefore, it is likely that the inhibition of autophagy is to enhance cell death induced by chaetocin.

### Chaetocin inhibits the growth of HepG2 xenografts

**Figure 8:**
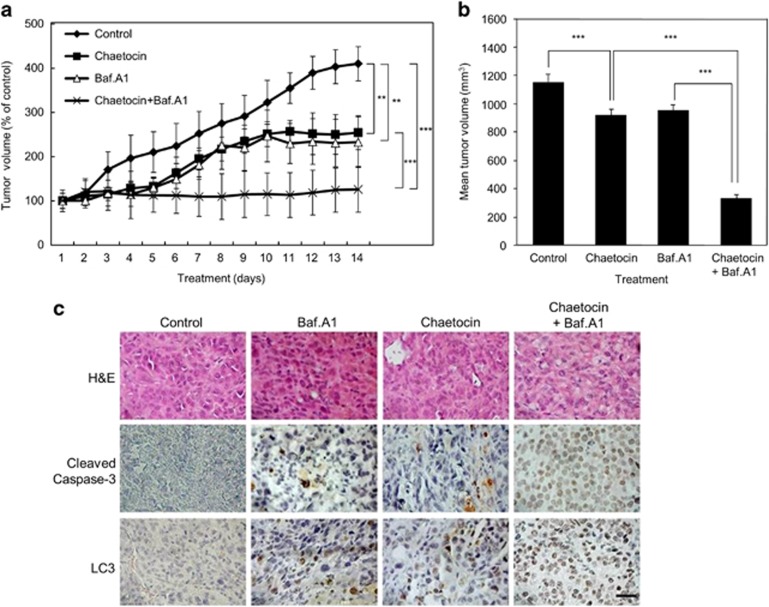
Chaetocin inhibits the growth of HepG2 xenografts. (**a**) Tumor volume of HepG2 xenografts with various treatments (chaetocin 0.25 mg/kg and Baf.A1 0.5 mg/kg) injected IP as indicated over 14 days. Shown are average tumor volumes (*n*=6) from NSG mice examined. (**b**) Mean tumor volume comparison for each treatment groups at day 14. Data are expressed as mean tumor volume±S.E.M. for pooled data from six mice (**P<0.005, ***P<0.001, by ANOVA). (**c**) Immunohistochemical analysis of cleaved caspase-3, LC3 and p21 on HepG2 tumor xenografts derived from (**a**). Representative images are shown at × 400 magnification. Scale bar, 10 *μ*m

In order to validate *in vitro* effects of chaetocin *in vivo*, tumor xenograft assays were performed. HepG2 cells (3 × 10^6^) were injected subcutaneously into NOD-scid IL2Rgamma^null^ mice (Figure 8a). Tumors were grown until they reached a volume of 100 mm^3^ before being injected intraperitoneally (IP) with PBS, chaetocin (0.25 mg/kg), Baf.A1 (0.5 mg/kg) or in combination with both chaetocin and Baf.A1 for 2 weeks. Tumors from the control group reached a volume of 1100 mm^3^ in 14 days, whereas the tumors receiving chaetocin alone or Baf.A1 alone were significantly smaller. More importantly, almost complete growth inhibition was observed in tumors receiving a combination of chaetocin and Baf.A1 (Figure 8b). Upon harvesting the tumors from each group, immunohistochemical staining was performed (Figure 8c). Tumors receiving chaetocin alone, Baf.A1 alone or in combination showed an increased staining for cleaved caspase-3 and LC3 compared with control tumors (Figure 8c). Moreover, the expression of cleaved caspase-3 and LC3 was stronger in combined treatment of chaetocin and Baf.A1 compared that of chaetocin alone or Baf.A1 alone.

## Discussion

Chaetocin is a fungal metabolite that is known to possess a potent antiproliferative activity in solid tumors by inducing cell death. There is growing body of evidence suggesting chaetocin's role in multiple biological pathways such as apoptosis, oxidative stress and proinflammatory response that influence the fate of cancer cells. However, the underlying molecular mechanisms inducing cell death has not clearly been elucidated. In this study, we have demonstrated for the first time that chaetocin induces autophagy in human hepatoma cells. We have shown that chaetocin is not only able to induce apoptosis, but also elicits autophagy. Moreover, we found that inhibiting autophagy resulted in the enhancement of cheatocin-mediated cell death. Autophagy is one of the key mechanisms regulating the turnover and recycling of cytoplasmic organelles as an adaptation response to stress.^15^ We have demonstrated that chaetocin results in the accumulation of LC3-II levels and increased GFP-LC3 puncta in HCC cell lines indicating that chaetocin is able to induce autophagy in HCC cells.

In order to extend the role of chaetocin-inducing autophagy, several cancer cell lines from various organ types, including those from the liver, brain and breast, were examined in our study. Chaetocin was able to readily induce autophagy in all the cell lines examined. These data suggest that the role of chaetocin as an autophagic inducer in the HCC can be extended to other cancer cell lines. Of course this does not eliminate the possibility that chaetocin may not induce autophagy in other cell lines not tested in this study, according to the study by Isham *et al.*, A549 cells treated with chaetocin in combination with autophagy inhibitors, 3-methyladenine or SP600125 did not affect chaetocin-induced apoptosis. This could be due to the differences in the cell's response to chaetocin suggesting that there may be a cell type specificity that dictates the efficacy of chaetocin in eliciting apoptosis.

Chaetocin has been shown to influence histone methylation by acting as an inhibitor of histone methyltransferases.^16^ A recent study by Chiba *et al.*^10^ demonstrated that chaetocin was able to reduce the levels of both H3K9me3 and histone lysine methyltrasnferase SUV39h1 in HCC. Consistent with our results, this study also observed chaetocin-mediated cell growth inhibition and the induction of apoptosis. This study presented data suggesting that chaetocin can be effectively used to inhibit histone methyltrasferase SUV39h1. Although the molecular mechanism by which chaetocin-mediated alteration in the H3K9me3 affects growth inhibition and induction of apoptosis has not been determined by this group, it is tempting to speculate that autophagy induced by chaetocin is mediated by its effect on epigenetic changes. As H3K9me3 is an important repressive histone mark in gene silencing, further analysis on gene expression following chaetocin treatment will likely contribute to the understanding of the molecular actions of chaetocin.

Previous studies have indicated that autophagy inhibition enhanced the efficacy of anticancer drugs, and suppression of autophagy may be an effective therapeutic strategy for cancer treatment.^17, 18, 19, 20^ We have therefore examined the link between apoptosis and autophagy in chaetocin-treated HCC cells and found that chaetocin-mediated apoptosis was enhanced upon loss of ATG5 or autophagy inhibitor treatment indicating that chaetocin-mediated autophagy promotes cell survival. Also the ability of chaetocin to attenuate HIF-1*α* activity and thereby inhibit angiogenesis in a HCC xenograft model^7^ makes chaetocin an even more attractive treatment strategy. In conclusion, we anticipate that combined treatment with chaetocin and autophagy inhibitors will offer an effective therapy for cancer treatment.

## Materials and Methods

### Cell lines and reagents

HepG2, Hep3B and Huh7 cells were cultured in DMEM medium containing 10% fetal bovine serum (Hyclone, Waltham, MA, USA), penicillin and streptomycin. Chaetocin was purchased from Enzo Life Sciences (Farmingdale, NY, USA). The pan-caspase inhibitor z-VAD-fmk was purchased from R&D Systems (Minneapolis, MN, USA). Baf.A1 was purchased from LC Laboratories (Woburn, MA, USA). Rapamycin was purchased from Calbiochem (La Jolla, CA, USA).

### Immunoblotting

Anti-procaspase-3, anti-cleaved caspase-3, anti-PARP and anti-ATG5 antibodies were purchased from Cell Signaling Technology (Beverly, MA, USA). Anti-LC3 (Medical and Biological Laboratories, Nagoya, Japan) antibodies were used at a dilution of 1 : 1000. Anti-*β*-actin antibody (Sigma Aldrich, St. Louis, MO, USA) was used at a dilution of 1 : 5000. Western blotting was performed as described previously.^21^ Immunoblotting was detected by enhanced chemiluminescence (Pierce, Rockford, IL, USA). The membrane was then exposed to X-ray film.

### Viable cell counting assay

Cells were seeded in six-well plates with a density of 3–5 × 10^5^ cells per well. After 18 h, they were treated with various concentration of chaetocin (0–1000 nM) for 24 h. After treatment, cells were detached from each well using 0.25% trypsin/EDTA. Trypan blue was then added to the cell suspension. The viable cell numbers were counted using a hemocytometer.

### Flow cytometric analysis of cell cycle

For flow cytometric analysis of DNA content, approximately 10^6^ cells were fixed in 80% ethanol at 4 °C for 24 h. Ethanol-fixed cells were stained with PI staining solution (50 *μ*g/ml PI, 0.1 mg/ml RNase A, 0.1% NP-40, 0.1% trisodium citrate) for 30 min and analyzed by a FACS analyzer (Becton-Dickinson Co., San Jose, CA, USA).

### Detection of LC3 translocation

For the analysis of green fluorescent protein-fused LC3 (GFP-LC3) localization, HepG2 cells, grown on two-well chamber, were transfected with GFP-LC3 plasmid using Lipofectmine 2000 (Invitrogen, Carlsbad, CA, USA). The GFP-LC3 plasmid was provided by Professor Tamotsu Yoshimori (Department of Cellular Regulation Research, Institute for Microbial Diseases, Osaka University, Japan).^22^ After 24 h, the medium was changed with complete medium, and positive stable clones were selected by growing cells added G418 (1 mg/ml) for 2 weeks. Stable GFP-LC3 transfected HepG2 cells were grown on two-well chamber slides and treated with 800 nM of chaetocin for 24 h. Cells were washed with PBS three times and fixed at room temperature for 10 min with 4% paraformaldehyde. After washing with PBS three times, cells were mounted with fluorescent mounting medium (Dako, Glostrup, Denmark). Images were obtained using a fluorescence microscope (Leica DM 3000, Wetzlar, Germany).

### Autophagic degradation assay

Degradation of long-lived protein was determined as described previously.^23^ Briefly, cells were incubated in normal culture medium containing [^14^C] leucine (0.5 *μ*Ci/ml) (Perkin Elmer, Waltham, MA, USA) for 24 h. The radioactive medium was removed, and the cells were incubated for 2 h in complete medium supplemented with 2 mM unlabeled leucine. The cells were washed once with PBS and treated with fresh medium containing 500 nM chaetocin, 20 nM Baf.A1 and 200 nM rapamycin for 4 h, respectively. The medium was precipitated in 10% trichloroacetic acid (TCA). The cells were washed with PBS, and 1 ml cold 10% TCA was added to each culture well for 5 min to fix the cellular proteins. Cells were then solubilized with 1 ml of 1 N NaOH. Radioactivity was measured by using a liquid scintillation counter (LSC) (Model LSC6500; Beckman, Brea, CA, USA). The degradation rate of long-lived radioactive proteins was calculated as the percentage of the radioactivity in the TCA-soluble supernatant relative to the total cellular radioactivity.

### Caspase-3/7 activity assay

To evaluate the activity of caspase-3/7, 5 × 10^4^ cells were seeded in each well of 96-well plate before being treated with chaetocin for 8 h. At the end of incubation, 100 *μ*l of Caspase-Glo 3/7 reagent (Promega, Madison, WI, USA) was added and luminescence detected using a Victor^3^ multilabel counter (Perkin Elmer Life and Analytical Sciences, Boston, MA, USA).

### RNA interference with siRNA

siRNAs against ATG5 and scrambled siRNA were obtained from Santa Cruz Biotechnology (Dallas, TX, USA). HepG2 cells were seeded in six-well plates at a density of 5 × 10^5^ cells per well and transfected with 150 nM siRNA using Lipofectamine 2000 (Invitrogen). After 24 h, the medium was changed to a complete growth medium, and cells were treated with chaetocin for the next 16 h. Cells were then harvested for cell viability and subjected to western blot analysis.

### Colony formation assay

HepG2 cells were seeded at 4 × 10^5^ cells in six-well culture plates and treatment with chaetocin and Baf.A1 for 24 h. The medium was replaced every 2–3 days with fresh medium for 14 days. The medium was discarded and the cells were washed with PBS twice. After fixing cells with 100% cold methanol for 10 min, cells were then stained with 1% crystal violet for 10 min.

### Tumor xenograft assays

For tumor formation *in vivo*, cells (3 × 10^6^/250 *μ*l) in equal volume of growth factor-reduced matrigel (BD Biosciences, Bedford, MA, USA) were injected subcutaneously into both flanks of 6-week-old male NOD-scid IL2Rgamma^null^ (NSG) mice (The Jackson Laboratory, Bar Harbor, ME, USA) as described previously.^24^ Mice were randomly assigned to different experimental groups: 0.25 mg/kg IP injection every day for 2 weeks of chaetocin in PBS; 0.5 mg/kg IP once a day for 2 weeks of Baf.A1 in PBS or combined treatment of chaetocin + Baf.A1. Tumors were measured daily, and the experiment was terminated at day 14 with tumors excised and weighed.

### Immunohistochemistry

Tumors were fixed in 10% formalin, embedded in paraffin and cut into 4 *μ*m sections. Deparaffinized and hydrated sections were blocked with 5% normal goat serum for 30 min, followed by incubation with primary antibodies at 4 °C overnight. Samples were washed three times with PBS and subsequently incubated with secondary antibody at room temperature for 30 min. Sections were incubated with DAB (Vector Laboratories, Burlingame, CA, USA) and were counterstained with hematoxylin and eosin before images being captured using a Carl Zeiss microscope (Carl Zeiss, Jena, Germany).

### Statistical analysis

Data represents mean±S.D. from three independent experiments. Statistical analysis was performed by Student's t-test or by ANOVA at a significance level of *P<0.05, **P<0.005 and ***P<0.001.

## Abbreviations

ATG5: autophagy protein 5
LC3-II: microtubule-associated protein light chain 3-II
PARP: poly ADP ribose polymerase
