# Supplementary figures and images for: The anticancer effect of chaetocin is enhanced by inhibition of autophagy
Source: Cell Death Dis. 2016 Feb 18;7(2):e2098–. doi: 10.1038/cddis.2016.15 (PMC5399187; doi:10.1038/cddis.2016.15)

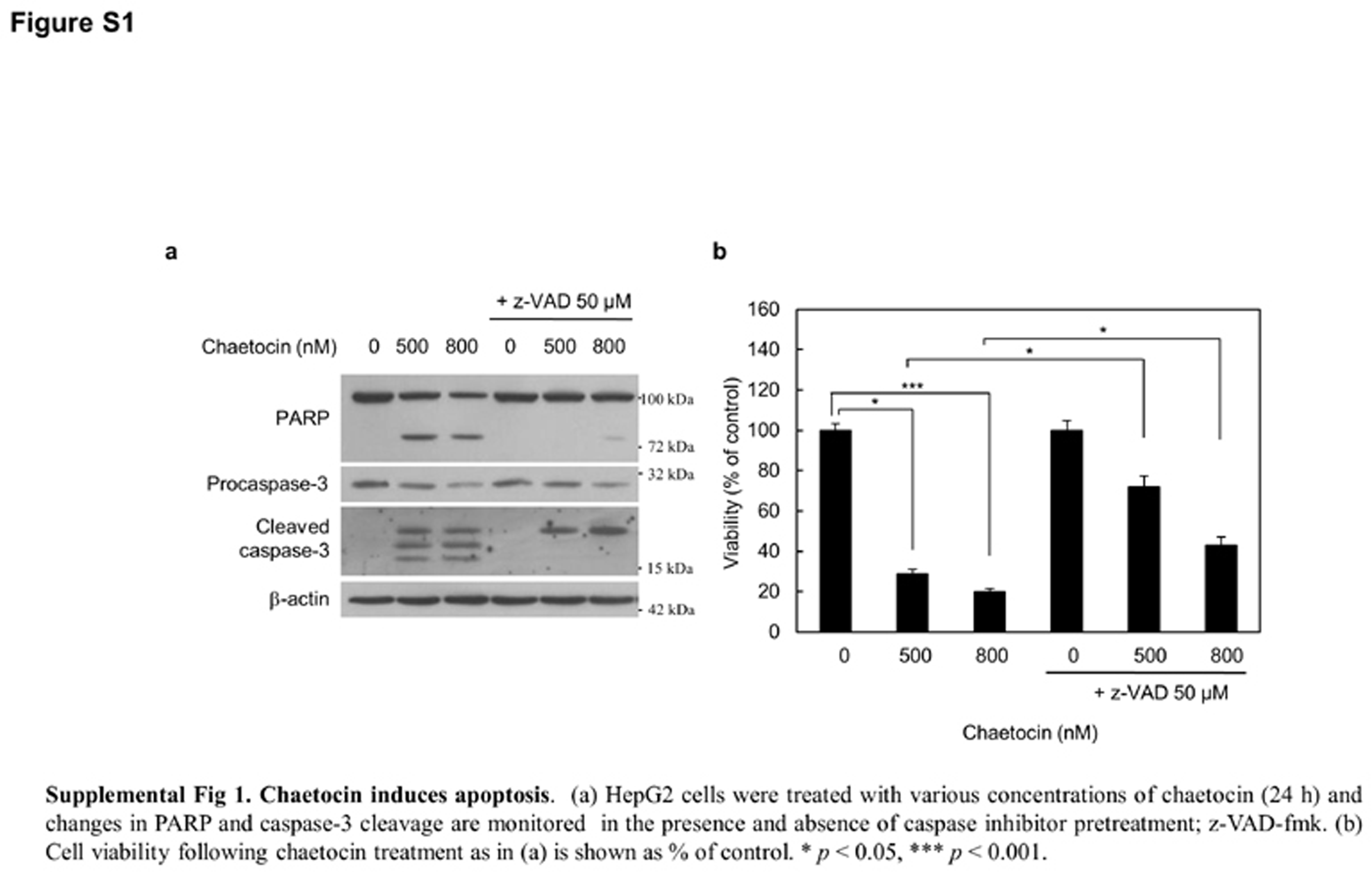

Supplement: Supplementary Figure 1 [file cddis201615x1.tif]

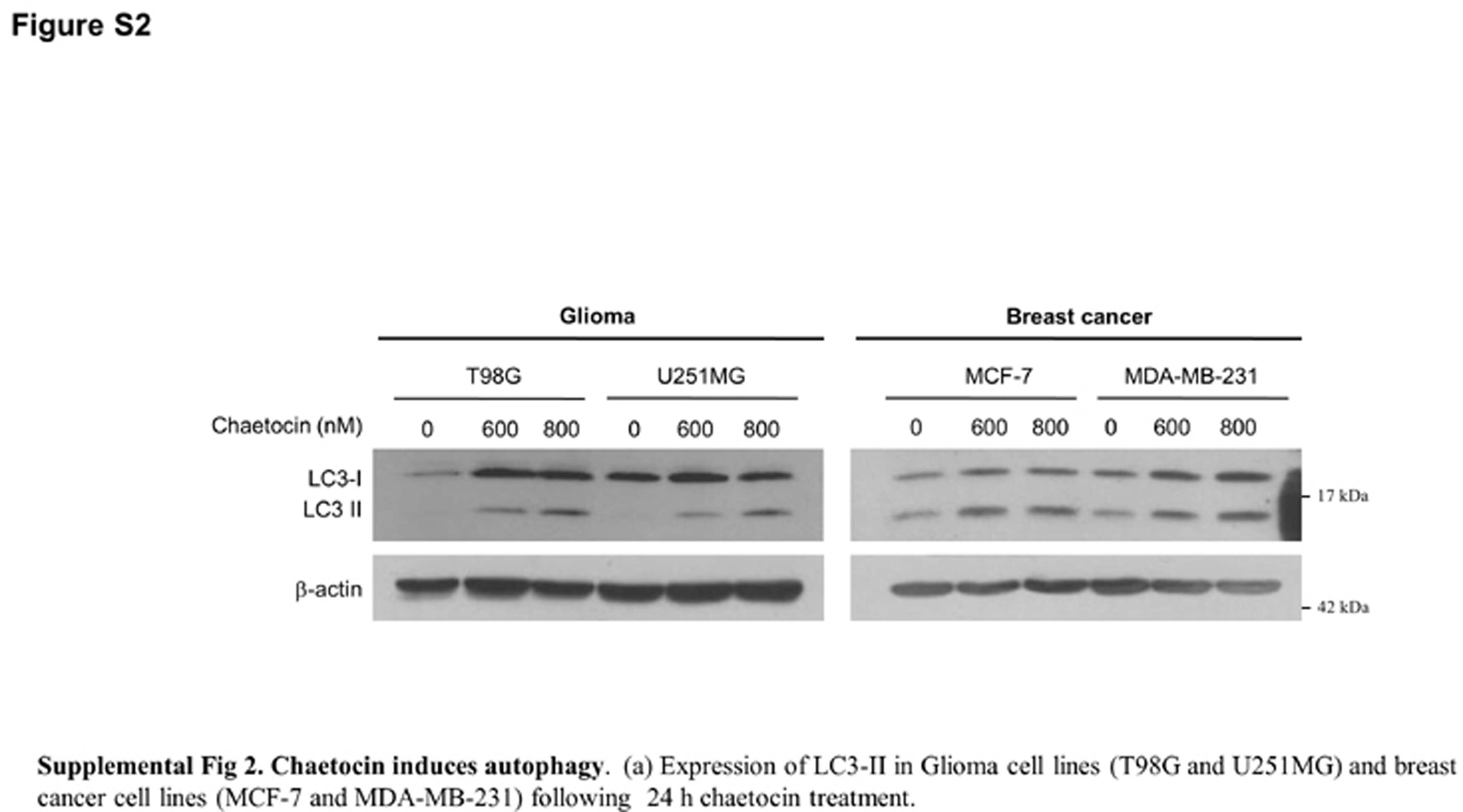

Supplement: Supplementary Figure 2 [file cddis201615x2.tif]

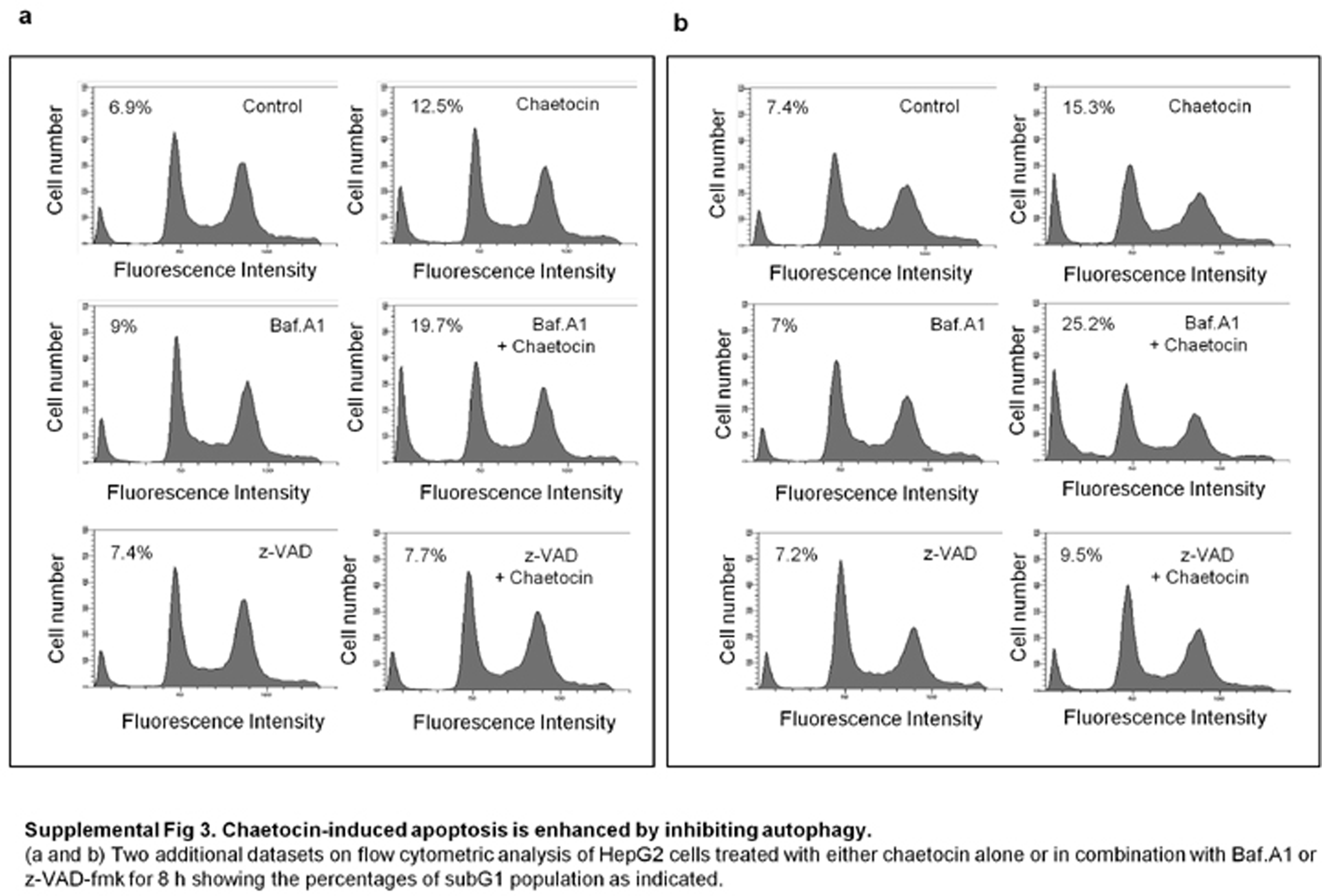

Supplement: Supplementary Figure 3 [file cddis201615x3.tif]

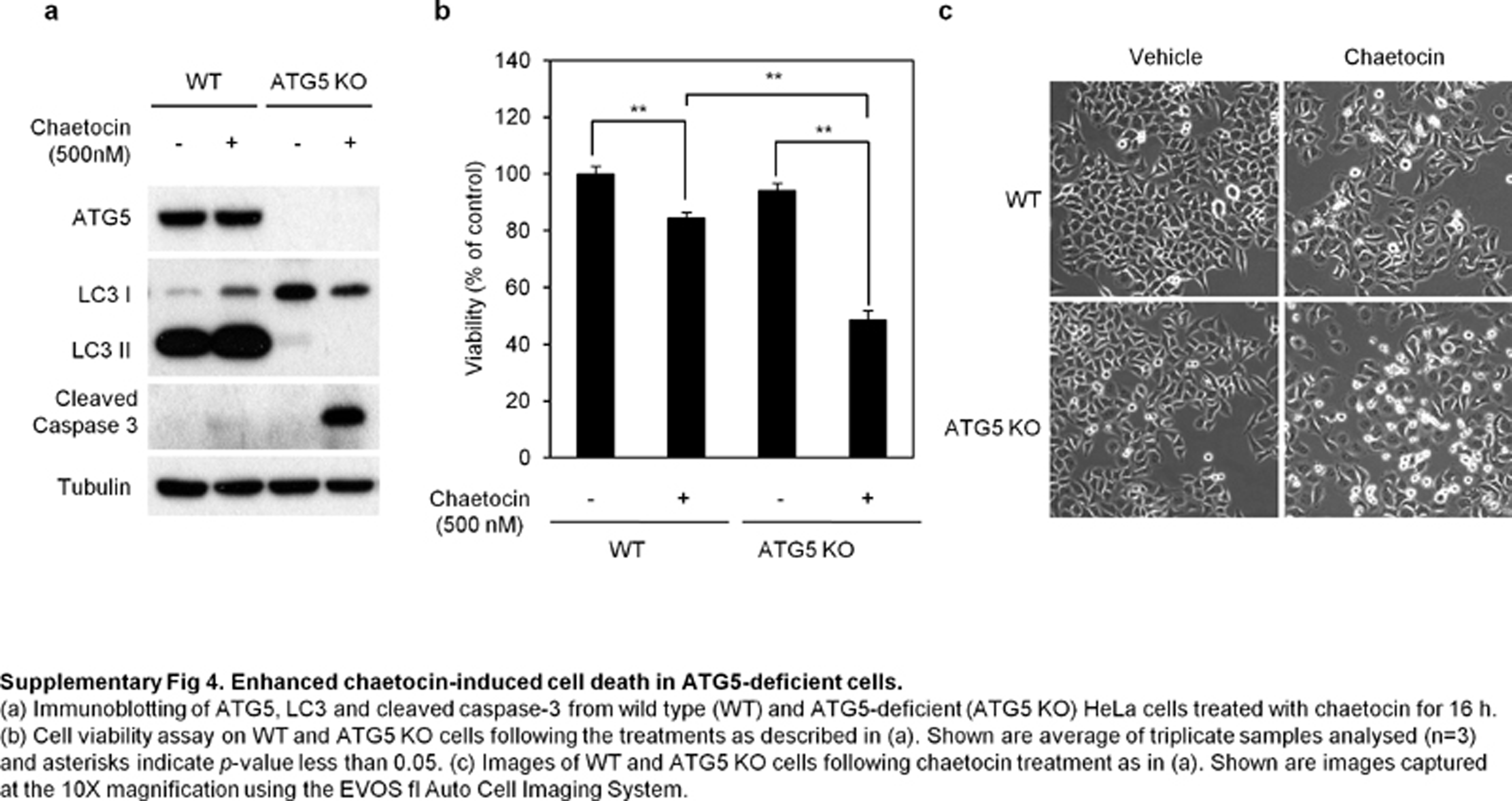

Supplement: Supplementary Figure 4 [file cddis201615x4.tif]
